# Subunit Vaccine Consisting of Multi-Stage Antigens Has High Protective Efficacy against *Mycobacterium tuberculosis* Infection in Mice

**DOI:** 10.1371/journal.pone.0072745

**Published:** 2013-08-15

**Authors:** Qi Xin, Hongxia Niu, Zhi Li, Guoping Zhang, Lina Hu, Bingxiang Wang, Jingjing Li, Hongjuan Yu, Wanbo Liu, Yue Wang, Zejiao Da, Ruiying Li, Qiaoyang Xian, Yong Wang, Ying Zhang, Tao Jing, Xingming Ma, Bingdong Zhu

**Affiliations:** 1 Lanzhou Center for Tuberculosis Research & Institute of Pathogenic Biology, School of Basic Medical Sciences, Lanzhou University, Lanzhou, China; 2 Lanzhou Institute of Biological Products, Lanzhou, China; 3 ABSL-3 Lab, Wuhan University, Lanzhou, China; 4 Department of Molecular Microbiology and Immunology, Bloomberg School of Public Health, Johns Hopkins University, Baltimore, United States of America; Fundació Institut d’Investigació en Ciències de la Salut Germans Trias i Pujol. Universitat Autònoma de Barcelona. CIBERES, Spain

## Abstract

To search for more effective tuberculosis (TB) subunit vaccines, antigens expressed in different growth stages of *Mycobacterium tuberculosis* (*M. tuberculosis*), such as RpfE (Rv2450c) produced in the stage of resuscitation, Mtb10.4 (Rv0288), Mtb8.4 (Rv1174c), ESAT6 (Rv3875), Ag85B (Rv1886c) mainly secreted by replicating bacilli, and HspX (Rv2031c) highly expressed in dormant bacilli, were selected to construct six fusion proteins: ESAT6-Ag85B-MPT64_190-198_-Mtb8.4 (EAMM), Mtb10.4-HspX (MH), ESAT6-Mtb8.4, Mtb10.4-Ag85B, ESAT6-Ag85B, and ESAT6-RpfE. The six fusion proteins were separately emulsified in an adjuvant composed of N,N’-dimethyl-N, N’-dioctadecylammonium bromide (DDA), polyribocytidylic acid (poly I:C) and gelatin to construct subunit vaccines, and their protective effects against *M. tuberculosis* infection were evaluated in C57BL/6 mice. Furthermore, the boosting effects of EAMM and MH in the adjuvant of DDA plus trehalose 6,6'-dimycolate (TDM) on BCG-induced immunity were also evaluated. It was found that the six proteins were stably produced in *E. coli* and successfully purified by chromatography. Among them, EAMM presented the most effective protection against *M. tuberculosis*. Interestingly, the mice that received EAMM+MH had significantly lower bacterial counts in the lungs and spleens than the single protein vaccinated groups, and had the same effect as those that received BCG. In addition, EAMM and MH could improve BCG-primed protective efficacy against *M. tuberculosis* infection in mice. In conclusion, the combination of EAMM and MH containing antigens from both replicating and dormant stages of the bacilli could induce robust immunity against *M. tuberculosis* infection in mice and may serve as promising subunit vaccine candidate.

## Introduction

*Mycobacterium tuberculosis* (*M. tuberculosis*) is a highly successful pathogen that is notorious for its ability to establish latent infection, which could serve as the source of new tuberculosis (TB) cases as we treat the existing cases [1,2]. More than two billion individuals are latently infected with this pathogen and about 10% of these people will eventually progress to active TB when their immune status declines [3], making it one of the top three infectious diseases globally. The increasing emergence of drug resistant TB, especially the multidrug-resistant (MDR) TB [4] and the extensively drug-resistant (XDR) TB [5,6], have made the fight to eliminate TB more difficult. In addition, co-infection with HIV exacerbates TB epidemic globally [7,8].

Bacillus Calmette–Guérin (BCG), the currently available vaccine against TB, has been used in humans since 1921 [9]. However, its efficacy in preventing TB remains controversial. BCG appreciably protects newborn babies and children from meningeal and miliary TB [10,11], but shows variable protection against adult pulmonary TB, ranging from 0% to 80% [12]. The high incidence of the disease at 8-9 million cases a year is at least partly a reflection of these problems. Hence, novel vaccines and vaccination strategy with the aim to control adult TB, especially latent TB infection are urgently needed.

At present, there are two major categories of potential vaccine candidates against *M. tuberculosis*. One is BCG replacement vaccines, including live recombinant BCG (rBCG) [13], attenuated mutants of *M. tuberculosis*, as well as modified non-pathogenic mycobacteria ( *M* *. vaccae* , RUTI [14], and *M. smegmatis* [15]) and the other is booster vaccines, such as fusion protein subunits, viral-vectored candidates (MVA85A, Ad35-TBS) [16,17], inactivated whole cell [18], whole cell lysates, and naked DNA vaccines [19]. One major reason for the failure of BCG in adults is that the protective immune responses induced by BCG inoculation at infant waned as children grow up. For this reason, it is believed that using subunit vaccine consisting of key antigens of *M. tuberculosis* as a booster vaccine in adolescence or adulthood to restore the immune response is a reasonable and practical vaccination strategy [20].

In the past few years, the TB subunit vaccine research field has made substantial progress, with several vaccines in clinical trials [21] (eg. MVA85A, Phase IIb; AREAS402, Phase IIb; Hybrid-I+IC31, Phase II; M72+AS01, Phase II; Hyvac 4/AERAS-404+IC31, Phase I; H56+IC31, Phase I; ID93+GLA-SE, Phase I). However, except the H56 [22] and ID93 [23] vaccines that consist of both replication stage and dormancy-related antigens, most subunit vaccines are based on antigens expressed in replicating stage, which may limit their protective efficacy. During *M. tuberculosis* infection, including initial infection stage, active TB and latent TB, tubercle bacilli population consists of growing, slow-growing and non-growing subpopulations with various metabolic states in a continuum and the subpopulations can interconvert to each other [24]. Therefore, ideal subunit vaccines, either prophylactic or therapeutic, should target all mycobacterial subpopulations.

In this study, in order to develop effective multi-stage tuberculosis subunit vaccines, antigens mainly expressed by the bacilli in various metabolic stages were selected to construct fusion proteins. We chose antigen RpfE (resuscitation promoting factor E), which was secreted from slowly replicating bacteria during reactivation as dormant bacteria resume active metabolism [25–27]; Mtb10.4 [28], Mtb8.4 [29], ESAT6 [30], the 190-198 peptide of MPT64, and Ag85B [31,32], which are extracellular proteins expressed by replicating bacilli; HspX, as part of the DosR regulon, which is a dormancy-related protein and accumulated in dormant mycobacteria during long-term survival in the host. These secreted antigens are strongly immunogenic and capable of providing strong protective immunity against *M. tuberculosis* challenge, suggesting that they are promising antigen candidates [28,33,34]. In this study, we constructed six novel fusion proteins without affinity tag, ESAT6-Ag85B-MPT64_190-198_-Mtb8.4 (EAMM), Mtb10.4-HspX (MH), ESAT6-Mtb8.4, Mtb10.4-Ag85B, ESAT6-Ag85B, and ESAT6-RpfE. Their protective efficacies were evaluated in a mouse model of *M. tuberculosis*. Our study showed that EAMM plus MH provided the highest protective efficacy against *M. tuberculosis* among the different protein subunit vaccine candidate.

## Materials and Methods

### Ethics statement

Animal experiments were conducted in compliance with the guidelines of China Council on Animal Care and Use. All animal procedures performed in this study were reviewed, approved, and supervised by the Institutional Animal Care and Use Committee of Gansu College of Traditional Chinese Medicine (permit number: SYXK(Gan) 2011-001). Animals received free access to water and commercial mouse chow throughout the study. During the experiments, the vaccinated and infected mice were monitored every day. Mice were sacrificed by cervical dislocation.

The experiment involving human participants was approved by the Human Research Ethics Committee of Lanzhou University and Lanzhou Pulmonary Hospital. Each individual was introduced the nature of the research and given the study protocol, and they all signed the informed consents.

### Animals and Human Subjects

Eight-week-old specific pathogen free 18- to 20-g female C57BL/6 mice were purchased from Slaccas Inc. (Shanghai, China) and kept at the animal facility under specific pathogen free conditions in Gansu College of Traditional Chinese Medicine. *M. tuberculosis* challenge experiments were performed at ABSL-3 lab at Wuhan University.

Blood cells from three human groups were used to analyze the immune activity in this study. They included TB patients, healthy close contacts (who may latently infected with *M. tuberculosis*), healthy BCG-vaccinated individuals. The group of patients consisted of 23 patients with pulmonary cavitary TB, 4 patients with pleural TB and 1 patient with miliary TB diagnosed with sputum or pleural fluid smear and culture, and chest X-ray. All TB patients were recruited from Lanzhou Pulmonary Hospital, Lanzhou, China. The group of close contacts consisted of eleven healthy nurses who had close contact with smear-positive pulmonary TB patients for more than five years in Lanzhou Pulmonary Hospital. Ten BCG vaccinated healthy students were recruited from Lanzhou University. IFN-γ responses to the *M. tuberculosis*-specific antigens ESAT-6, CFP-10 were analyzed in these subjects.

### Construction, expression and purification of the fusion proteins

The coding sequences of desired genes were PCR-amplified from the genomic DNA of *M. tuberculosis* H37Rv, and the generated fragments were directionally inserted into the corresponding multiple cloning sites of pET30a (+) to construct plasmids encoding the six fusion proteins respectively. The sequences of the DNA inserts were verified by sequencing.

The recombinant plasmids were transformed into the *E. coli* strain BL21 (DE3), and the expression of fusion proteins were induced by isopropyl β-D-thiogalactopyranoside (IPTG). After induction, cells were harvested by centrifugation and were lysed by sonication. Samples were sedimented by centrifugation at 10,000g for 10 min at 4°C to separate inclusion body proteins and soluble proteins. The overexpressed EAMM, Mtb10.4-Ag85B, ESAT6-Ag85B, and ESAT6-RpfE aggregated into inclusion bodies, while MH and ESAT6-Mtb8.4 remained in supernatant in soluble form. The inclusion bodies were dissolved in Solubilization buffer (8 M urea and 50 mM Tris; PH 8.0) at a ratio of 1: 10 w/v for 30 min at 4°C separately; then they were refolded by gradient dialysis for 60h at 4°C. Successive chromatographic purification steps were used in the purification of the recombinant proteins using AKTA Purifier 100 (GE Healthcare, Piscataway, NJ). The purification of EAMM was performed by one-step ion-exchange chromatography (IEX) on a DEAE Sepharose Fast Flow column using buffer A (20 mM Tris, pH 7.5) and buffer B (20 mM Tris and 1M NaCl, pH 7.5) as gradient eluent. Two-step successive chromatography was used in the purification of Mtb10.4-Ag85B, ESAT6-Ag85B, ESAT6-RpfE and ESAT6-Mtb8.4. The protein samples were first loaded on DEAE Sepharose Fast Flow column and step-eluted by buffer C (20 mM phosphate buffer; pH 7.4) and buffer D (20 mM phosphate buffer, 1M NaCl; pH 7.4). Then the peak fractions of the target proteins were pooled, and further purified by a hydrophobic chromatography (HIC) on Butyl Sepherose High Performance column. MH was purified by three successive chromatographic purification steps [35]. The molecular mass and purity of these purified proteins were assessed by 12% (v/v) SDS-PAGE.

### Cultured ELISPOT in human PBMCs

Human PBMCs were prepared by centrifugation with Human Lymphocyte Separation Tube (Dakewe Biotech Company Ltd., Shenzhen, China). Human IFN-γ ELISPOT kits (Dakewe Biotech Company Ltd., Shenzhen, China) were performed to detect IFN-γ secretion following the manufacturer’s instruction. PBMCs (3×10^5^ cells/well) were cultured in 96-well microtiter plates precoated with anti-human IFN-γ and stimulated with either single antigen HspX (10 µg/ml), ESAT-6 (20 µg/ml) or fusion protein MH (10 µg/ml), EAMM (20 µg/ml). In addition, PBMCs were cultured with medium alone or with PHA (1 µg/ml) as negative or positive control, respectively. The spots were counted using an ELISPOT reader (Bio-sys, GmbH, Karben, Germany) and results were expressed as the mean of spot-forming cells per 3×10^5^ cells ± standard deviation (SD).

### Immunogenicity and protective efficacy of the fusion proteins

Mice were immunized three times at 3-week intervals with subunit vaccines containing 20 µg of EAMM, MH, ESAT6-Mtb8.4, Mtb10.4-Ag85B, ESAT6-Ag85B, ESAT6-RpfE or 10 µg EAMM plus 10 µg MH emulsified in an adjuvant composed of DDA (250 µg/dose; Sigma-Aldrich, Poole, UK), poly(I:C) (50 µg/dose; Sigma-Aldrich, Poole, UK) and gelatin (0.4% W/V; which was added to stabilize the formulations) or with a single dose of BCG. For BCG immunization, mice were inoculated with 5×10^6^ CFU BCG at the time of the first subunit vaccination. BCG and all antigens were applied by subcutaneous (s.c.) route with a total volume of 200 µl/mouse.

At 6 week after the last immunization, animals were sacrificed for immunogenicity detection using ELISPOT assay following 20-h incubation of cells with 10 µg/ml special antigens, such as ESAT-6, Ag85B, Mtb8.4, Mtb10.4_1-18_ peptide, HspX or RpfE. Splenocytes (6×10^5^ cells/well) were cultured in microtiter plates precoated with anti-mouse IFN-γ. The ELISPOT was performed according to manufacturer’s protocol. The spots were counted as described above and results were expressed as the mean spot-forming cells per 6 ×10^5^ cells ± SD.

Ten weeks after the last immunization, mice were challenged with 5×10^5^ CFU of *M. tuberculosis* H37Rv by caudal vein injection. Six weeks later, the animals were sacrificed, and the tissues (lung and spleen) were homogenised in sterile saline and plated at 10-fold serial dilutions on Middlebrook 7H11 selective agar (BD, NJ, USA) enriched with OADC (BD, NJ, USA). Agar plates were incubated at 37°C and colony forming Units (CFUs) were enumerated 21 days later. Protection results were shown in log_10_ (mean CFU) ± SD.

### Protective immunity and efficacy of EAMM and Ag85B-MPT64_190-198_-Mtb8.4 (AMM) as boosters of BCG

The BCG - primed mice were boosted with subunit vaccine EAMM or AMM [36] twice at 12 and 14 week after BCG prime. EAMM or AMM (20 µg/dose) was emulsified in adjuvant composed of DDA (250 µg/dose) and trehalose 6,6'-dimycolate (TDM) (50 µg/dose). For BCG immunization, mice were inoculated subcutaneously with 5×10^6^ CFU BCG at 0 week.

Splenocytes were isolated from mice at 6 week after the last boosting to determine the relative number of IFN-γ or IL-17 expressing cells. Mouse IFN-γ and IL-17 ELISPOT kits (U-Cytech BV, Utrecht, the Netherlands) were used to analyze the cytokines production according to the instruction manual. Briefly, the spleen cells (5×10^5^ cells/well) were incubated for 48-h with antigens (ESAT-6, 8 µg/ml; Ag85B, 5 µg/ml; PPD, 10 µg/ml) in microtiter plates. The spots were counted as described above. Mice were challenged with *M. tuberculosis* H37Rv strain 10 weeks after the last vaccination and the CFUs of *M. tuberculosis* in the lungs and spleens were enumerated as described above.

### Pulmonary histopathological analysis and detection of acid-fast bacilli

For histopathological analysis and detection of acid-fast bacilli in the lungs of *M. tuberculosis* infected mice, the tissues were fixed, embedded, sectioned and stained as described previously [35,36].

### Statistics analysis

Results were presented as mean ± SD. Variance was analyzed by one-way ANOVA using SPSS 13.0 software. The results were considered statistically significant for *p* < 0.05.

## Results

### Construction and purification of the six novel fusion proteins as subunit vaccine candidates

**Figure 1:**
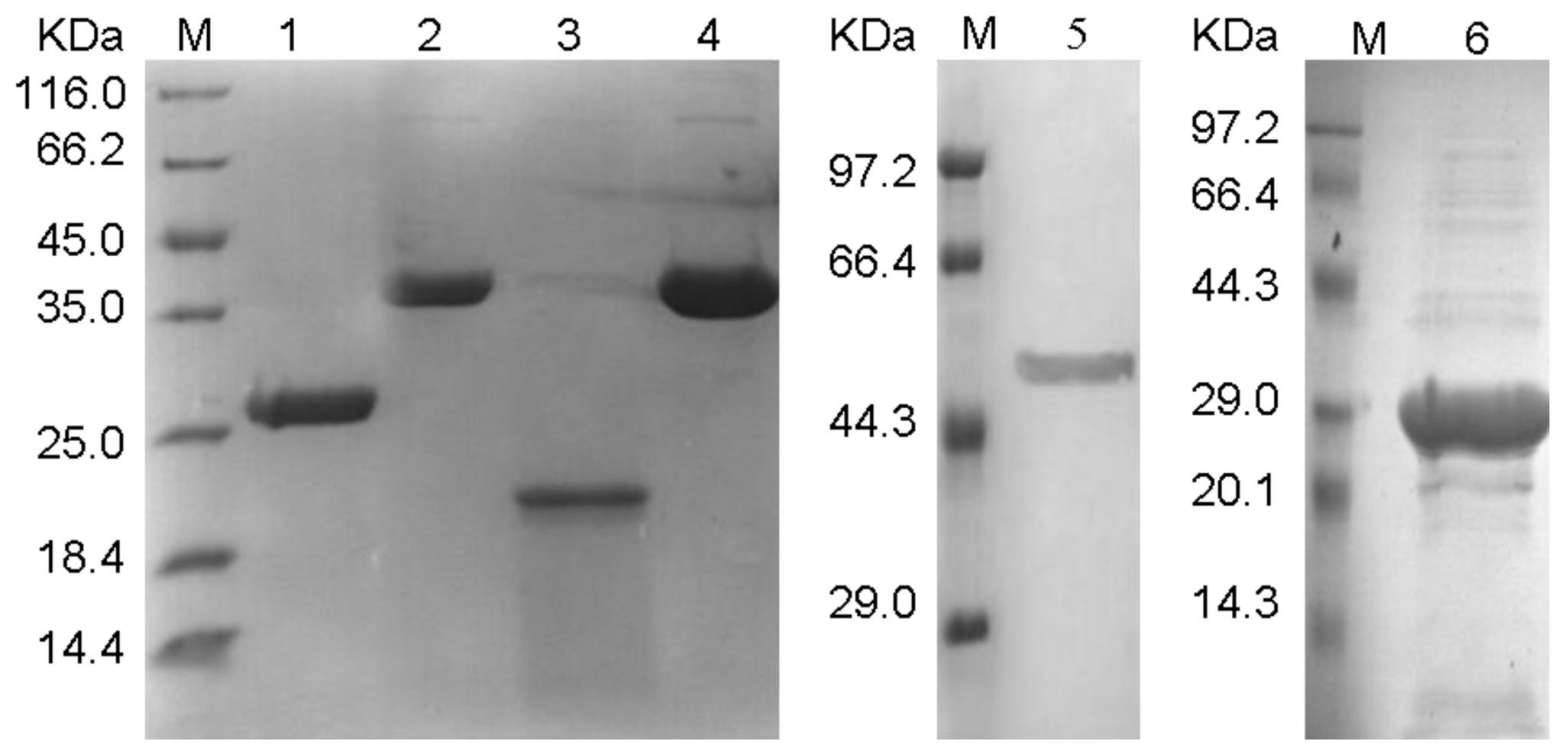
Purified fusion proteins were visualized in Coomassie-stained gels. Lane 1, Purified MH; Lane 2, Purified ESAT6-Ag85B; Lane 3, Purified ESAT6-Mtb8.4; Lane 4, Purified Mtb10.4-Ag85B; Lane 5, Purified EAMM; Lane 6, Purified ESAT6-RpfE.

The recombinant plasmids expressing *M. tuberculosis* antigens RpfE, Mtb10.4, Mtb8.4, ESAT6, Ag85B, Mtb8.4 and HspX were constructed with His-tag. The fusion proteins EAMM, MH, ESAT6-Mtb8.4, Mtb10.4-Ag85B, ESAT6-Ag85B, and ESAT6-RpfE were efficiently expressed in *E. coli* BL21 (DE3) respectively. Among these recombinant proteins, EAMM [37], Mtb10.4-Ag85B, ESAT6-Ag85B, ESAT6-RpfE were expressed in inclusion bodies and were purified by one-step or two-step chromatography, whereas MH [35,38] and ESAT6-Mtb8.4 were soluble and mainly expressed in the supernatant and were purified by successive chromatography. The purified fusion proteins were shown to be free of any significant amount of *E. coli* contaminating proteins on SDS-PAGE and migrated with their expected molecular weight (Figure 1).

## Immunogenicity and Protective Efficacy of the Fusion Proteins in Mice

**Figure 2:**
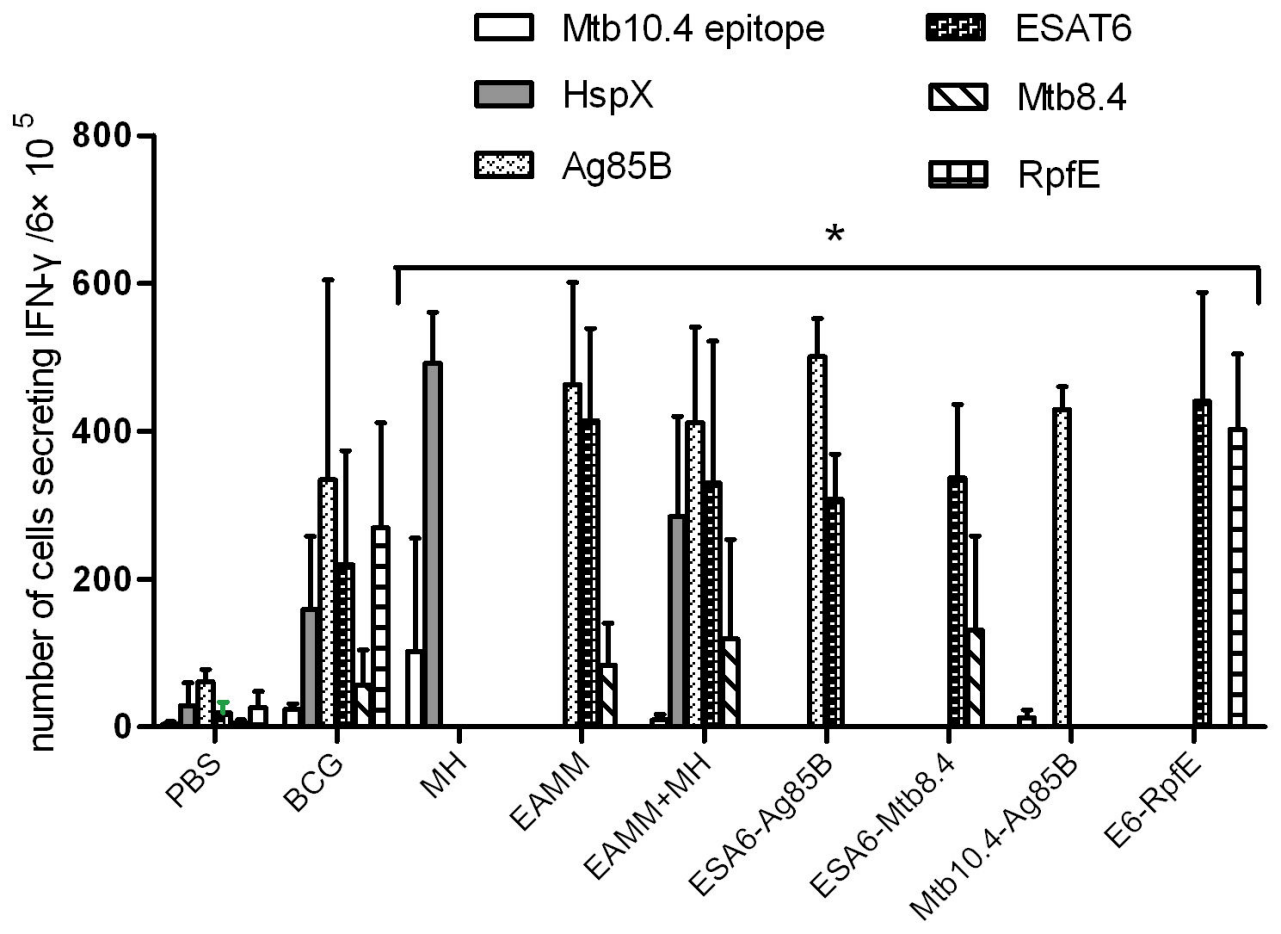
Antigen specific IFN-γ secretion in splenocytes of mice immunized with different vaccine candidates. Mice were immunized three times (3 weeks apart) with 20 µg of subunit vaccines formulated in DDA/Poly(I:C)/Gelatin or with a single dose of BCG (5×10^6^ CFU). Six weeks after the final vaccination, spleen cells were stimulated with specific antigens separately and the IFN-γ releasing cells were assayed by ELISPOT. Results are presented as mean ± SD from groups of four mice. * *p* < 0.05 vs. PBS and BCG groups.

C57BL/6 mice were immunized three times with the fusion proteins emulsified in the adjuvant of DDA/Ploy(I:C)/Gelatin. The mice were sacrificed 6 weeks after the last inoculation, and the secretion of IFN-γ in spleen lymphocyte was evaluated *in vitro* by ELISPOT with the stimulation of specific antigens or epitopes. The results showed that the levels of IFN-γ secreted by spleen cells from mice immunized with the protein vaccines were higher than that from the PBS control (*p* < 0.05), but there was no obvious difference among the six protein vaccine candidates (Figure 2).

**Figure 3:**
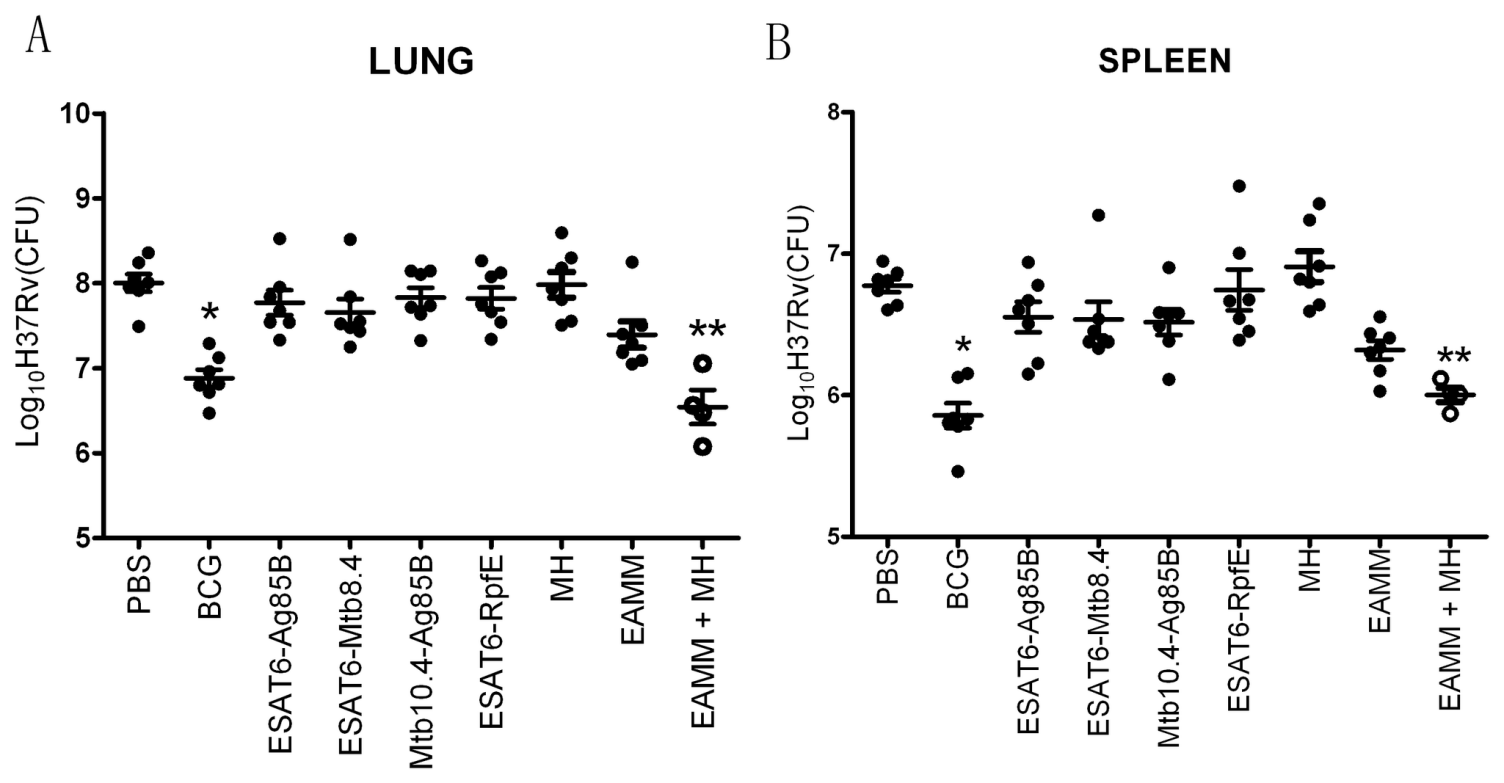
Protective efficacy of subunit vaccine candidates against *M. tuberculosis* infection. The mice received three times of inoculation with the protein vaccine candidates in DDA/Poly(I:C)/Gelatin. The vaccinated mice were challenged with virulent *M. tuberculosis* 10 weeks after the final vaccination. Six weeks after challenge with virulent *M. tuberculosis*, the animals were sacrificed, and the numbers of CFU of *M. tuberculosis* in the lungs (A) and spleens (B) were determined. Results are presented as mean ± SD, *n* = 4 or 7. *p* < 0.05 *vs*. PBS. ** *p* < 0.05 vs. PBS, EAMM and MH.

As for the protective effects, EAMM showed the most effective protection to contain bacterial growth among the fusion proteins, reducing approximately 0.6 log_10_ bacterial counts. MH, which contains the dormant antigen HspX, did not show any protection. But it is interesting that MH combined with EAMM significantly reduced bacterial counts in the lungs (1.5 ± 0.13 log_10_ CFU reduction) and spleens (0.8±0.12 log_10_ CFU reduction) compared with PBS group (*p* < 0.05), and was as effective as BCG (Figure 3A, 3B).

### IFN-γ profile in response to EAMM and MH in human PBMCs

**Figure 4:**
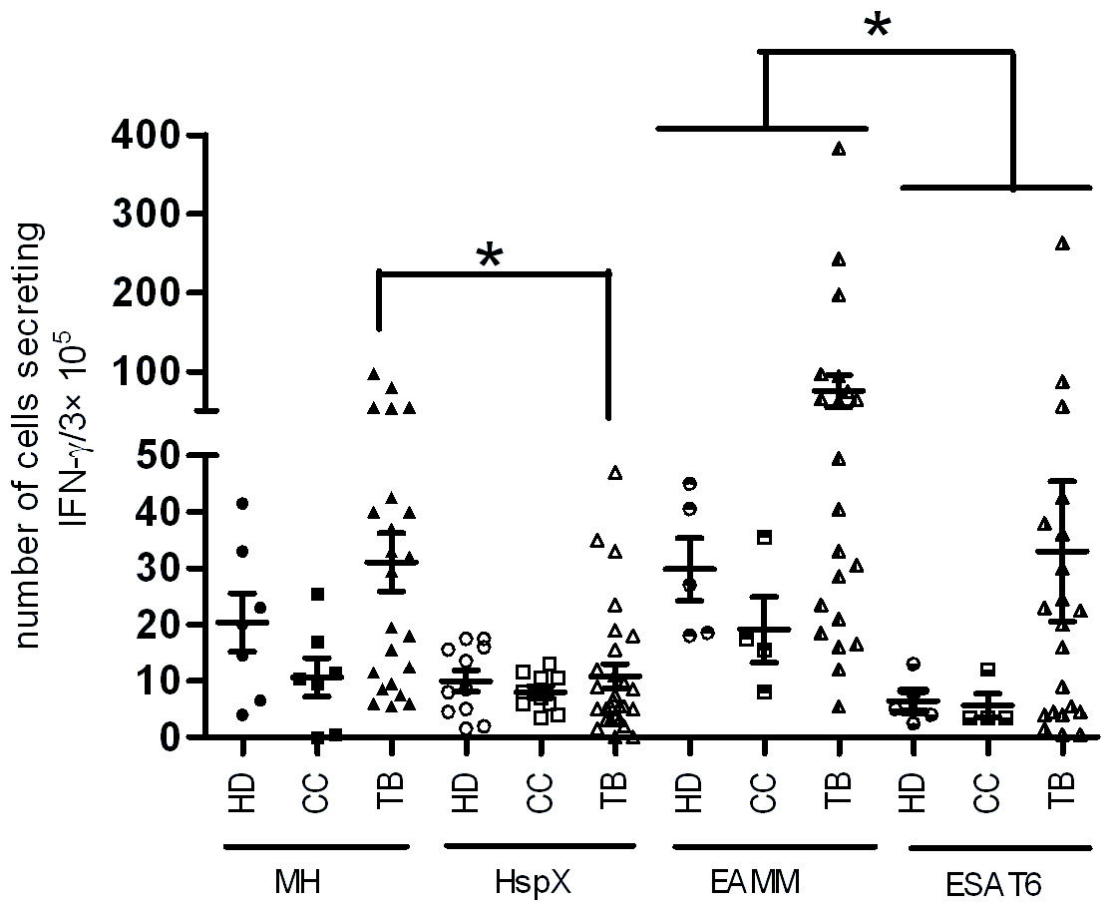
IFN-γ response profiles to recombinant proteins MH, HspX, EAMM and ESAT-6 in different groups of donors. Healthy BCG-vaccinated donors (HD, *n* = 5-11), close contacts (CC, *n* = 4-10) and active TB patients (TB, *n* = 21-28) were recruited. IFN-γ secreting cells were analyzed using human IFN-γ ELISPOT kits. Freshly isolated PBMCs from these subjects were plated in duplicate at 3 ×10^5^ cells per well in 96 spot and incubated with the recombinant proteins (10 µg/ml or 20 µg/ml) for 48 h at 37°C. Data are shown as mean ± SD. * *p* < 0.01.

PBMCs from 28 TB patients, 11 close contacts and 10 healthy BCG vaccinated controls were co-cultured with recombinant proteins separately and the secreted IFN-γ was measured. EAMM stimulated higher numbers of IFN-γ secreting T cells in all three groups than ESAT6 (*p* < 0.01) (Figure 4). MH also stimulated higher level of IFN-γ than HspX, especially in TB patients (*p* < 0.01). These results indicate that fusion proteins composed of multiple antigens could induce higher antigen specific immune responses than single antigen in humans.

### Boosting BCG with EAMM and AMM in adjuvant of DDA-TDM

**Figure 5:**
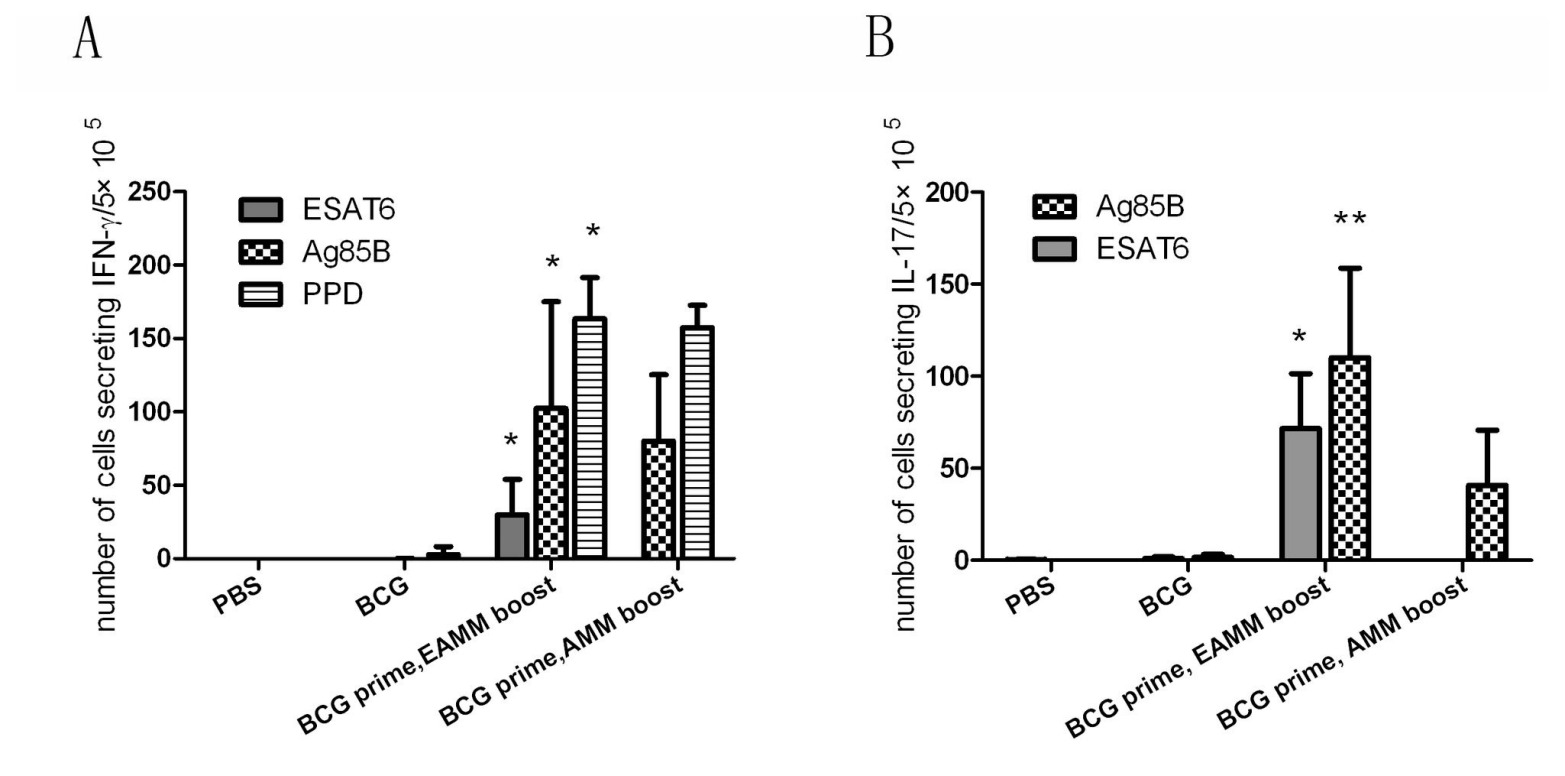
IFN-γ and IL-17 secretion in splenocytes of mice primed with BCG and boosted with subunit vaccine candidates. C57BL/6 mice were primed with BCG, and boosted twice with EAMM or AMM emulsified in the adjuvant of DDA/TDM by s.c. at 12 and 14 week after prime. Six weeks after the last boosting, spleen cells were stimulated with purified protein derivative of tuberculin (PPD), ESAT6 or Ag85B *in vitro* respectively, and IFN-γ and IL-17 were assayed by ELISPOT. (A) IFN-γ secretion in splenocytes. (B) IL-17 secretion in splenocytes. Results are presented as mean ± SD, *n* = 4. * *p* < 0.05 vs. PBS and BCG groups;. ** *p* < 0.05 vs. PBS, BCG and BCG/AMM groups.

Six weeks after EAMM and AMM boosting, lymphocytes of vaccinated mice were cultured in the presence of special antigens and analyzed by ELISPOT for the frequencies of IFN-γ and IL-17 secreting cells (Figure 5A, 5B). With the stimulation of specific antigens, the frequencies of IFN-γ and IL-17 secreting lymphocytes from the mice primed with BCG and boosted with EAMM or AMM were higher than that immunized with BCG without boosting (*p* < 0.05). Our previous study also showed that boosting BCG-immunized mice with MH resulted in higher IFN-γ and IL-17 expression following the stimulation of Mtb10.4_1–18_ peptide, HspX, and PPD, compared with BCG alone (*p* < 0.05) [35].

**Figure 6:**
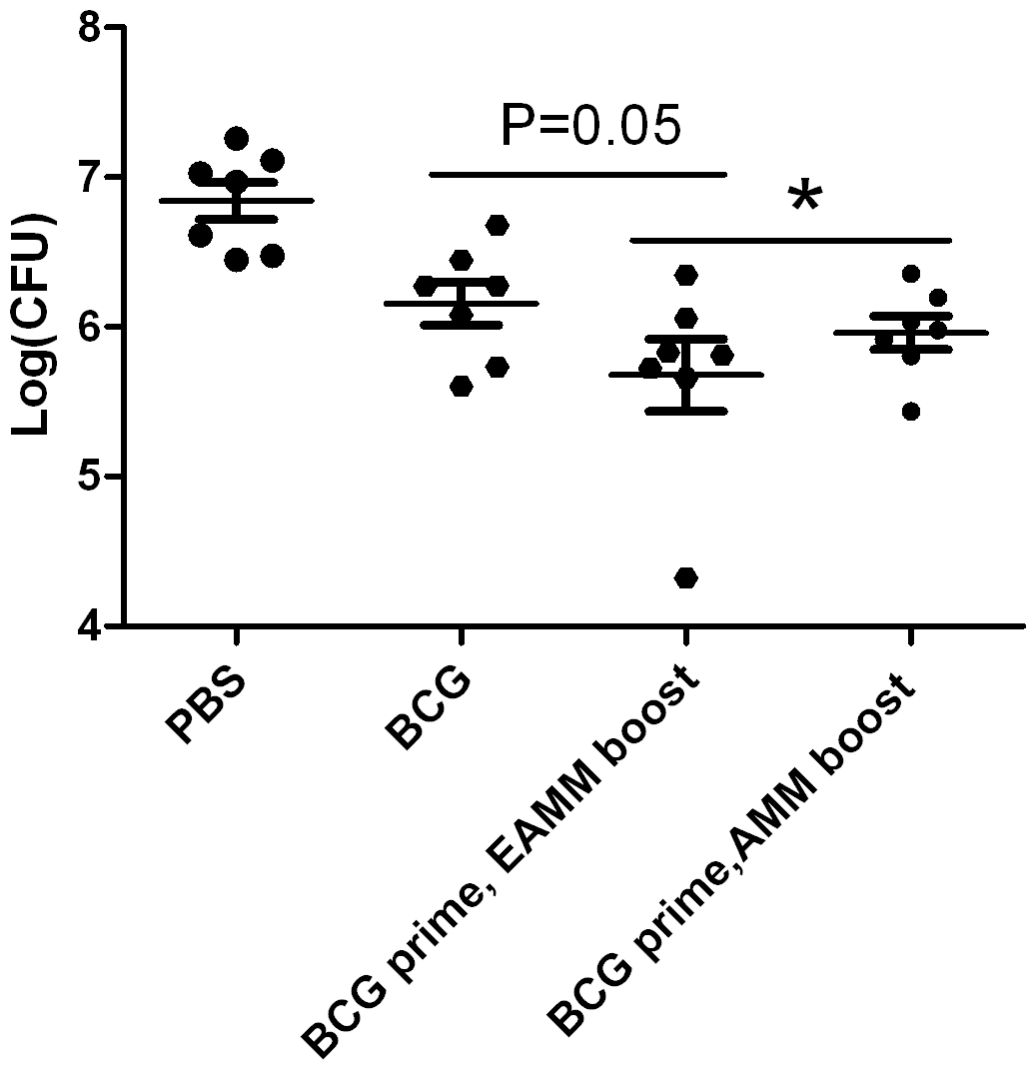
Protective efficacy against virulent *M. tuberculosis* H37Rv infection in mice primed with BCG and boosted with EAMM or AMM. Mice were primed with BCG and boosted by EAMM/AMM two times at the 12th and 14th week. Ten weeks after boosting, mice were challenged with 5×10^5^ CFU of *M. tuberculosis* H37Rv. Six weeks after challenge the bacterial load in the lungs was determined. Results are presented as mean ± SD, *n* = 7. *p < 0.05 vs. PBS.

To determine the protective efficacy of EAMM, AMM and MH as boosters of BCG, the mice were challenged with virulent *M. tuberculosis* H37Rv 10 weeks after the last vaccination, and the bacterial counts in the lungs were enumerated six weeks after challenge. In comparison to PBS-vaccinated control, mice boosted with all these three subunit vaccines had lower bacterial counts (*p* < 0.05) (Figure 6). Among them, EAMM-boosting resulted in a 1.17 ± 0.32 log_10_ CFU reduction compared with PBS control, lower than BCG vaccine (0.69 ± 0.06 log_10_ CFU reduction) (*p* = 0.05), AMM-boosting (0.88 ± 0.012 log_10_ CFU reduction) and MH-boosting [35] (1.09 ± 0.08 log_10_ CFU reduction). These findings indicate that among these immunized groups EAMM boost induced the best protection against *M. tuberculosis* challenge.

**Figure 7:**
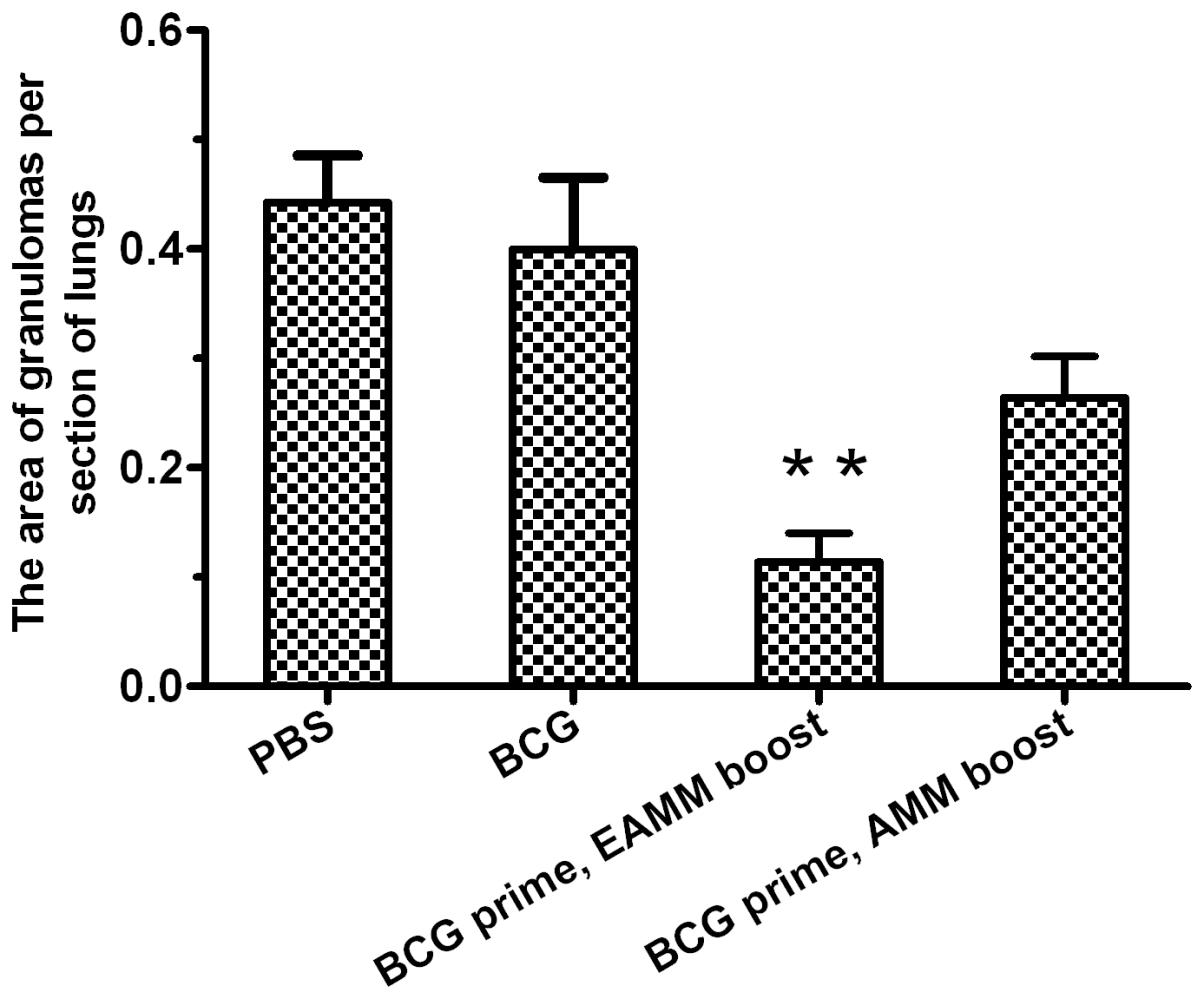
Histopathology induced by vaccine immunization and *M. tuberculosis* challenge. After infection, the upper left lobes of the lungs were harvested, fixed in 10% formalin and embedded in paraffin blocks, and then sectioned for light microscopy. Data represents the area ratio of granuloma in sections stained with HE. Seven sections of lungs were selected for each group. * *p* < 0.05 vs. PBS, BCG and AMM group.

Pulmonary histological examination of the challenged mice revealed that all vaccine immunizations had reduced lesions to some extent relative to the PBS control (*p* < 0.05, Figure 7). The mice primed with BCG and boosted with EAMM resulted in significantly less manifestation of lesions compared with the BCG and BCG/AMM groups (*p* < 0.05). The acid-fast *M. tuberculosis* bacilli were hardly present in the lung tissues of EAMM boosted mice (data not shown), which was consistent with the CFU results and lessened histopathological lesions.

## Discussion

In this study, we constructed six novel fusion proteins consisting of 190-198 peptide of MPT64 and antigens RpfE, Mtb10.4, Mtb8.4, ESAT6, Ag85B and HspX, which are expressed in various stages of bacterial life cycle, and analyzed their immunogenicity and protective efficacy. Our study showed that all fusion proteins EAMM, MH, ESAT6-Mtb8.4, Mtb10.4-Ag85B, ESAT6-Ag85B and ESAT6-RpfE could induce robust T cell responses in mice, but their protective efficacies varied. Among them, EAMM vaccine induced the strongest antigen specific immune responses in both humans and mice, and also provided the most effective protection against *M. tuberculosis* infection in mice while MH and other proteins did not show such significant protective effects. However, the combination of EAMM and MH vaccine showed even better protective efficacy than EAMM or MH alone. Boosting BCG-primed mice with EAMM also induced higher protective effect than AMM, MH, or without boost.

Our previous study had constructed the fusion protein AMM and proved that AMM had the potential to boost BCG primed immunity [36]. To improve the protective efficacy of AMM, ESAT-6 encoded by RD1 region of the *M. tuberculosis* genome which is absent from *M. bovis* BCG was linked together with AMM to construct a four-component fusion protein EAMM. AMM-boosted mice showed slightly lower CFUs (0.88 ± 0.012 log_10_ CFU reduction) than the BCG alone vaccinated mice (0.69 ± 0.06 log_10_ CFU reduction) (Figure 6). However, boosting BCG-primed mice with EAMM resulted in a 1.17 ± 0.12 log_10_ reduction of CFU in lungs compared with the PBS-immunized group, which was obviously lower than that provided by BCG vaccination alone (*p* = 0.05, Figure 6). These findings indicate that adding ESAT-6 enhanced the protective effect of AMM. It has been reported that restoration of RD1 promoted the capacity of BCG vaccination for protection against *M. tuberculosis* [39]. Vaccine based on fusion protein Ag85B-ESAT6 had been a subunit vaccine candidate that entered into clinical trials [40]. Our work showed that ESAT-6 incorporated into AMM effectively improved the protective efficacy of AMM. However, it is worth noting that in this study, the fusion protein ESAT6-Ag85B in adjuvant of DDA/Poly (I:C)/Gelatin showed only a slight protection (Figure 3A, 3B). But with the same adjuvant, we had much better protection against *M. tuberculosis* with EAMM than with ESAT6-Ag85B.

Up to now, our lab has evaluated 9 vaccine candidates based on 9 fusion proteins including AMM [36], Ag85B-MPT64_190-198_-HspX (AMH) [41], EAMM, MH [35], ESAT6-Ag85B, ESAT6-Mtb8.4, Mtb10.4-Ag85B, ESAT6-RpfE, and Ag85B. We found that EAMM provided the most effective protection against *M. tuberculosis* infection among them all (Figure 3A, 3B), followed by AMM which showed higher immunogenicity and protective efficacy than Ag85B alone [36]. The differences in protective effect among the fusion proteins might reflect that some antigens contain more immunodominant epitopes that could induce higher immune responses and higher protective effect. The same tendency was also observed in human PBMCs responses: EAMM stimulation induced stronger T cell responses than fusion protein MH, ESAT6, and HspX (Figure 4). In addition, multi-component vaccine might be beneficial for vaccination of genetically diverse human populations by decreasing the evasion induced by variations of antigen and antigen presentation [33].

The infecting bacilli, either at initial or late stage of infection, are heterogeneous and consist of at least three subpopulations of *M. tuberculosis*, including growing, slow-growing and non-growing bacilli [24]. Consequently, antigens in various growth stages should be included to construct novel multi-stage TB subunit vaccines so as to provide complete immune protection against bacteria in different metabolic states [42]. We found that mice receiving EAMM plus MH had significantly lower bacterial counts in the lungs and spleens compared with EAMM or MH alone. In addition, our previous study also found that fusion protein AMH containing dormancy-related antigen could enhance the effect of AMM [41]. MH and AMH alone did not confer obvious protective effect, but when they were combined with EAMM or AMM they improved the protective effect of EAMM and AMM significantly. We evaluated the protective efficacy of the multi-stage vaccine candidates in an active TB model which contains both proliferating and dormant bacteria [24]. The protective effect of them would be more obvious in latent TB infection model [22]. These findings suggest that multi-stage vaccines combining antigens from proliferating and dormant stages could generate broad and strong responses which would provide broad protective activity against *M. tuberculosis* infection*.*

Cell-mediated immune (CMI) response is believed to be the primary host protective response against *M. tuberculosis* infection [43,44]. It was demonstrated that IFN-γ produced from CD4+ T cells regulated the immunity against TB [45]. However, the level of IFN-γ alone cannot reflect vaccine protection against *M. tuberculosis* infection well. Recently, T helper 17 (Th17) cell, a new CD4 +T cell subset, has been identified [46]. Th17 cells produce IL-17 which is capable of inducing expression of chemokines, such as G-CSF, CXC chemokines, IL-6, and IL-8 that promote Th1 cell recruitment and granuloma organization throughout infection [47,48]. In addition, it was reported that, similar to IFN-γ, IL-17 could act as an effector molecule against *M. tuberculosis* infection to protect against human TB [49]. Besides producing IFN-γ, mice receiving BCG-prime and EAMM-boost generated robust antigen specific IL-17, while the MH vaccine-boosted mice generated less HspX-specific IFN-γ and IL-17 than EAMM-boost [35]. The production of IFN-γ and IL-17 are consistent with the protective efficacy of EAMM and MH as boosters of BCG.

In summary, we evaluated six fusion proteins as possible vaccine candidates and found that the fusion protein EAMM induced the strongest immune responses and protective efficacy among them all, and the combination of EAMM and MH showed higher protective efficacy than EAMM alone. Furthermore, we found that BCG-prime and EAMM/MH-boost could improve the protective efficacy of BCG. These findings suggest that the combination of EAMM and MH containing multiple antigens from both replicating and dormant *M. tuberculosis* may be a promising multi-stage subunit vaccine. Inbred mouse strains differ in a large number of genetic factors which may affect the presentation of antigen to T lymphocytes. Future studies are warranted to further evaluate the EAMM and MH vaccine candidate in other animal models including the BALB/c, rabbit, non-human primate model.
